# Supplementary material for: Genome Size Variation Assessment in Vitis vinifera L. Landraces in Ibiza and Formentera (Balearic Islands)
Source: Plants (Basel). 2022 Jul 21;11(14):1892. doi: 10.3390/plants11141892 (PMC9320920; doi:10.3390/plants11141892)
Supplement: Supplementary file 1 [file plants-11-01892-s001.zip › plants-1778436-supplementary.pdf]

SUPPLEMENTARY MATERIALS

Table S1: Results of Tukey's test showing statistically significant differences between means on all cultivars and landraces.

Cultivar/ Landrace (i)	Cultivar/ Landrace (j)	Average differences (i-j)	Error deviation	P< 0.05	Lower limit	Higher limit
AG2	Santa Magdalena	-0.1129	0.0279	0.014	-0.2140	-0.0118
AG1	Garnatxa	0.1395	0.0381	0.046	0.0012	0.2778
Santa Magdalena	AG2	0.1129	0.0279	0.014	0.0118	0.2140
Santa Magdalena	Valencí tinto/ Grumier	0.1655	0.0372	0.003	0.0308	0.3003
Santa Magdalena	Garnatxa	0.1872	0.0398	0.001	0.0430	0.3315
Danugue	Garnatxa	0.1501	0.0410	0.045	0.0016	0.2987
Valencí tinto/ Grumier	Santa Magdalena	-0.1655	0.0372	0.003	-0.3003	-0.0308
Garnatxa	AG1	-0.1395	0.0381	0.046	-0.2778	-0.0012
Garnatxa	Santa Magdalena	-0.1872	0.0398	0.001	-0.3315	-0.0430
Garnatxa	Danugue	-0.1501	0.0410	0.045	-0.2987	-0.0016

Table S2: Results of Tukey's test showing statistically significant differences on Ibizan cultivars and landraces.

Cultivar/ Landrace (i)	Cultivar/ Landrace (j)	Average differences (i-j)	Error deviation	P< 0.05	Lower limit	Higher limit
AG2	Santa Magdalena	-0.1138	0.0285	0.013	-0.2145	-0.0132
AG1	Valencí tinto/Grumier	0.1305	0.0365	0.044	0.0017	0.2592
Santa Magdalena	AG2	0.1138	0.0285	0.013	0.0132	0.2145
Santa Magdalena	Valencí tinto/Grumier	0.1655	0.0365	0.002	0.0367	0.2943
Danugue	Valencí tinto/Grumier	0.1909	0.0455	0.007	0.0303	0.3516
Valencí tinto/ Grumier	AG1	-0.1305	0.0365	0.044	-0.2592	-0.0017
Valencí tinto/ Grumier	Santa Magdalena	-0.1655	0.0365	0.002	-0.2943	-0.0367
Valencí tinto/ Grumier	Danugue	-0.1909	0.0455	0.007	-0.3516	-0.0303

Table S3: Results of Tukey's test showing statistically significant differences on Formentera cultivars and landraces.

Cultivar/ Landrace (i)	Cultivar/ Landrace (j)	Average differences (i-j)	Error deviation	P< 0.05	Lower limit	Higher limit
Moscatell	Garnatxa	0.1681	0.0421	0.013	0.0269	0.3094
Garnatxa	Moscatell	-0.1681	0.0421	0.013	-0.3094	-0.0269
Garnatxa	Quigat	-0.1420	0.0421	0.048	-0.2832	-0.0008
Quigat	Garnatxa	0.1420	0.0421	0.048	0.0008	0.2832

Table S4: Origin, voucher number, collectors and dates of the studied material.

Taxon (code)	Voucher number	Origin, collectors and date

Vitis vinifera L. 'Beba' (1)	BC-992593	Ibiza, Balearic Islands: private vineyard near Sant Agustí des Vedrà, González, 02 July 2018
Vitis vinifera L. 'Valencí tinto/Grumier'(2)	BC-992594	Ibiza, Balearic Islands: private vineyard near Sant Agustí des Vedrà, González, 02 July 2018
Vitis vinifera L. 'Moscatell' (3)	BC-992595	Ibiza, Balearic Islands: private vineyard near Sant Agustí des Vedrà, González, 02 July 2018
Vitis vinifera L. 'AG2' (5)	BC-992596	Ibiza, Balearic Islands: private vineyard near Sant Agustí des Vedrà, González, 02 July 2018
Vitis vinifera L. 'Teta de vaca' (6)	BC-992597	Ibiza, Balearic Islands: private vineyard near Sant Josep de sa Talaia, González, 31 July 2017
Vitis vinifera L. ´Beba' (7)*	BC-992598	Ibiza, Balearic Islands: private vineyard near Sant Josep de sa Talaia, González, 31 July 2017
Vitis vinifera L. 'Morzacà' (8)*	BC-992599	Ibiza, Balearic Islands: private vineyard near Sant Josep de sa Talaia, González, 31 July 2017
Vitis vinifera L. 'Grec' (9)*	BC-992600	Ibiza, Balearic Islands: private vineyard near Sant Josep de sa Talaia, González, 31 July 2017
Vitis vinifera L. 'AG1' (10)*	BC-992601	Ibiza, Balearic Islands: private vineyard near Sant Josep de sa Talaia, González, 31 July 2017
Vitis vinifera L. 'Sultanita' (11)	BC-992602	Ibiza, Balearic Islands: private vineyard near Sant Josep de sa Talaia, González, 31 July 2017
Vitis vinifera L. 'Fresa' (12)*	BC-992603	Ibiza, Balearic Islands: private vineyard near Sant Josep de sa Talaia, González, 31 July 2017
Vitis vinifera L. 'Santa Magdalena' (13)	BC-992604	Ibiza, Balearic Islands: private vineyard near Sant Josep de sa Talaia, González, 31 July 2017
Vitis vinifera L. 'Danugue' (14)	BC-992628	Ibiza, Balearic Islands: private vineyard near Ibiza town, González, 31 July 2017
Vitis vinifera L. 'Maçanet' (15)*	BC-992605	Ibiza, Balearic Islands: private vineyard near Ibiza town, González, 31 July 2017
Vitis vinifera L. 'Callet negrella' (16)	BC-992606	Ibiza, Balearic Islands: private vineyard near Ibiza town, González, 31 July 2017
Vitis vinifera L. 'Fogoneu' (17)	BC-992607	Ibiza, Balearic Islands: private vineyard near Ibiza town, González, 31 July 2017
Vitis vinifera L. 'Vermelleta' (18)*	BC-992608	Ibiza, Balearic Islands: private vineyard near Ibiza town, González, 31 July 2017
Vitis vinifera L. 'Grec' (19)*	BC-992609	Ibiza, Balearic Islands: private vineyard near Sant Josep de sa Talaia, González, 31 July 2017
Vitis vinifera L. 'Valencí tinto/Grumier' (20)	BC-992610	Ibiza, Balearic Islands: private vineyard near Sant Jordi de ses Salines, González, 31 July 2017
Vitis vinifera L. 'Santa Magdalena' (21)	BC-992611	Ibiza, Balearic Islands: private vineyard near Sant Josep de sa Talaia, González, 25 July 2017
Vitis vinifera L. 'AG2' (22)	BC-992612	Ibiza, Balearic Islands: private vineyard in Es Cubells, González, 02 July 2017
Vitis vinifera L. 'AG2' (23)	BC-992613	Ibiza, Balearic Islands: private vineyard in Es Cubells, González, 24 July 2017
Vitis vinifera L. 'AG1' (24)*	BC-992614	Ibiza, Balearic Islands: private vineyard in Es Cubells, González, 24 July 2017
Vitis vinifera L. 'Colló de gall' (25)*	BC-992615	Ibiza, Balearic Islands: private vineyard in Es Cubells, González, 24 July 2017

Vitis vinifera L. 'Beba' (26)	BC-992616	Ibiza, Balearic Islands: private vineyard near Es Cubells, González, 02 July 2017
Vitis vinifera L. 'Danugue' (27)	BC-992617	Ibiza, Balearic Islands: private vineyard in Es Cubells, González, 24 July 2017
Vitis vinifera L. 'AG1' (28)*	BC-992618	Ibiza, Balearic Islands: private vineyard in Es Cubells, González, 24 July 2017
Vitis vinifera L. 'AG1' (29)*	BC-992627	Formentera, Balearic Islands: private vineyard near Ca Marí, González, 26 July 2017
Vitis vinifera L. 'Manses tibbus' (30)	BC-992619	Formentera, Balearic Islands: private vineyard near Ca Marí, González, 26 July 2017
Vitis vinifera L. 'Garnatxa' (31)	BC-992620	Formentera, Balearic Islands: private vineyard near Ca Marí, González, 26 July 2017
Vitis vinifera L. 'Tinto velasco' (32)	BC-992621	Formentera, Balearic Islands: private vineyard near Ca Marí, González, 26 July 2017
Vitis vinifera L. 'AG2' (33)	BC-992622	Formentera, Balearic Islands: private vineyard near Ca Marí, González, 26 July 2017
Vitis vinifera L. 'Danugue' (34)	BC-992623	Formentera, Balearic Islands: private vineyard near Ca Marí, González, 26 July 2017
Vitis vinifera L. 'Grec' (35)*	BC-992624	Formentera, Balearic Islands: private vineyard near Ca Marí, González, 17 July 2018
Vitis vinifera L. 'AG1' (36)*	BC-992625	Formentera, Balearic Islands: private vineyard near Ca Marí, González, 17 July 2018
Vitis vinifera L. 'Quigat' (37)	BC-992626	Formentera, Balearic Islands: private vineyard near Cala Saona, González, 17 July 2018

*Ibiza and Formentera local landraces of *Vitis vinifera*. 'AG1' and 'AG2' are landraces that had not been catalogued previously therefore, they do not have a name yet.
Code (4) has not been considered in the study; we keep this accession for possible further work, but it had growth development issues and the informant was unsure of its origin and name.
