# Supplementary figures and images for: Genome Size Variation Assessment in Vitis vinifera L. Landraces in Ibiza and Formentera (Balearic Islands)
Source: Plants (Basel). 2022 Jul 21;11(14):1892. doi: 10.3390/plants11141892 (PMC9320920; doi:10.3390/plants11141892)

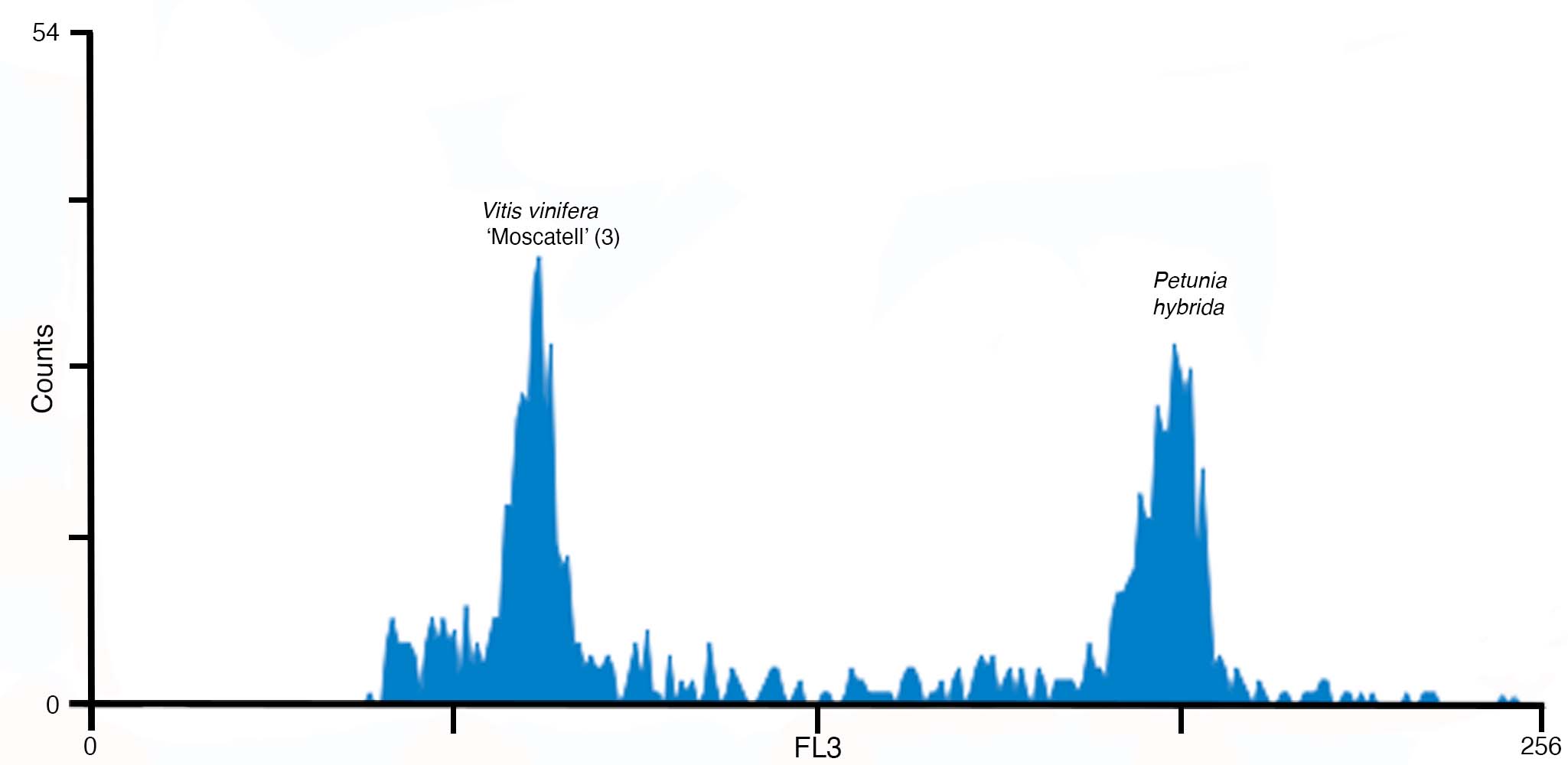

Supplement: Supplementary file 1 [file plants-11-01892-s001.zip › FigureS1.jpg]
